# Multiple Tuberculous Bronchopleural Fistulas Complicated by Hydropneumothorax: A Case Report of Successful Conservative Management

**DOI:** 10.7759/cureus.98205

**Published:** 2025-11-30

**Authors:** Mohamad Zikir Ismail, Fathin Hadi, Wan Aireene Wan Ahmed

**Affiliations:** 1 Internal Medicine, Universiti Sains Malaysia, Kubang Kerian, MYS; 2 Radiology, Hospital Universiti Sains Malaysia, Kubang Kerian, MYS

**Keywords:** antitubercular therapy, bronchopulmonary fistula (bpf), conservative management, hydropneumothorax, mycobacterium tuberculosis (mtb)

## Abstract

We report the case of a 61-year-old man with poorly controlled diabetes mellitus and a history of chronic smoking who presented with two months of fever, productive cough, weight loss, and night sweats. Initial chest radiography revealed a right cavitary lesion with hydropneumothorax, and pleural fluid analysis was positive for *Mycobacterium tuberculosis* by GeneXpert with markedly elevated adenosine deaminase. Despite catheter drainage and anti-tuberculosis therapy (ATT), a persistent pneumothorax prompted chest tube insertion. Computed tomography confirmed multiple bronchopleural fistulas, a rare complication of pulmonary tuberculosis usually requiring surgical management. Given his stable clinical condition, a conservative strategy was adopted. He improved on ATT with the gradual resolution of pneumothorax. He was discharged with a pneumostat and remained well at follow-up, with weight gain and no recurrence after completing six months of therapy. This case illustrates that selected patients with multiple tuberculous bronchopleural fistulas and hydropneumothorax can be successfully managed conservatively with close monitoring and effective ATT, avoiding the need for surgical intervention.

## Introduction

*Mycobacterium tuberculosis* causes tuberculosis (TB), which remains one of the most common infectious diseases worldwide, with millions of new cases reported each year despite global control efforts [1]. Pulmonary TB typically presents with cough, fever, and weight loss, but it may also lead to serious complications. Bronchopleural fistula (BPF) is one such complication, defined as an abnormal communication between the bronchial tree and the pleural space, resulting in persistent air leakage, pneumothorax, or empyema [2].

Tuberculous BPF is uncommon, and most reported cases describe a single fistula that often necessitates surgical intervention [3]. The occurrence of multiple tuberculous BPFs complicated by hydropneumothorax is extremely rare, with only limited documentation in the literature. While surgery is generally considered the standard approach, selected patients may improve with conservative treatment when infection is adequately controlled, and the clinical condition remains stable.

TB-related pleural complications, including pneumothorax, empyema, and BPF, substantially increase morbidity, especially in patients with extensive parenchymal disease or delayed diagnosis [4]. BPF formation in TB is thought to arise from progressive caseous necrosis extending to the pleural surface, creating a direct connection between affected bronchi and the pleural cavity [5]. Studies have shown that hydropneumothorax associated with tuberculous BPF can lead to prolonged air leak and risk of secondary infection, which often prompts early surgical evaluation [6].

Although surgical closure remains the recommended treatment for persistent or complicated BPF, recent reports suggest possibilities for successful conservative management in selected cases [5]. Adequate drainage, infection control, and timely anti-tuberculosis therapy (ATT) may facilitate spontaneous closure of the fistula, especially in clinically stable patients without respiratory compromise [5,7]. Several case reports have demonstrated resolution of tuberculous BPF with ATT and conservative measures alone, emphasising the role of individualised management strategies [5,8].

Here, we report a rare case of multiple tuberculous BPFs complicated by hydropneumothorax in a patient with poorly controlled diabetes and chronic smoking, successfully managed with ATT and conservative measures without the need for surgical intervention.

## Case presentation

A 61-year-old man with a 30-pack-year smoking history and poorly controlled type 2 diabetes mellitus with HbA1c of 11% presented with a two-month history of nocturnal fever, productive cough, anorexia, fatigue, and a 19 kg weight loss. He had no known TB contact or family history of malignancy. His symptoms persisted despite two courses of oral antibiotics from primary care clinics. On admission, he appeared cachectic and febrile, with reduced breath sounds over the right hemithorax. Laboratory investigations revealed leukocytosis and elevated C-reactive protein (Table 1).

**Table 1:** Laboratory result. Hb: hemoglobin; WBC: white blood cells; CRP: C-reactive protein; ESR: erythrocyte sedimentation rate; LDH: lactate dehydrogenase

Parameter	Value	Reference Range
WBC	13.2× 10^9^/L	3.8-10.6 × 10^9^/L
Hb	12.4 g/dL	13.3-16.3 g/dL
Platelet	480	165-415 × 10^9^/L
CRP	210 mg/L	<5 mg/L
ESR	87 mm/hr	0-10 mm/hr
Protein	72 g/L	66-87 g/L
LDH	301 U/L	135-225 U/L

**Figure 1:**
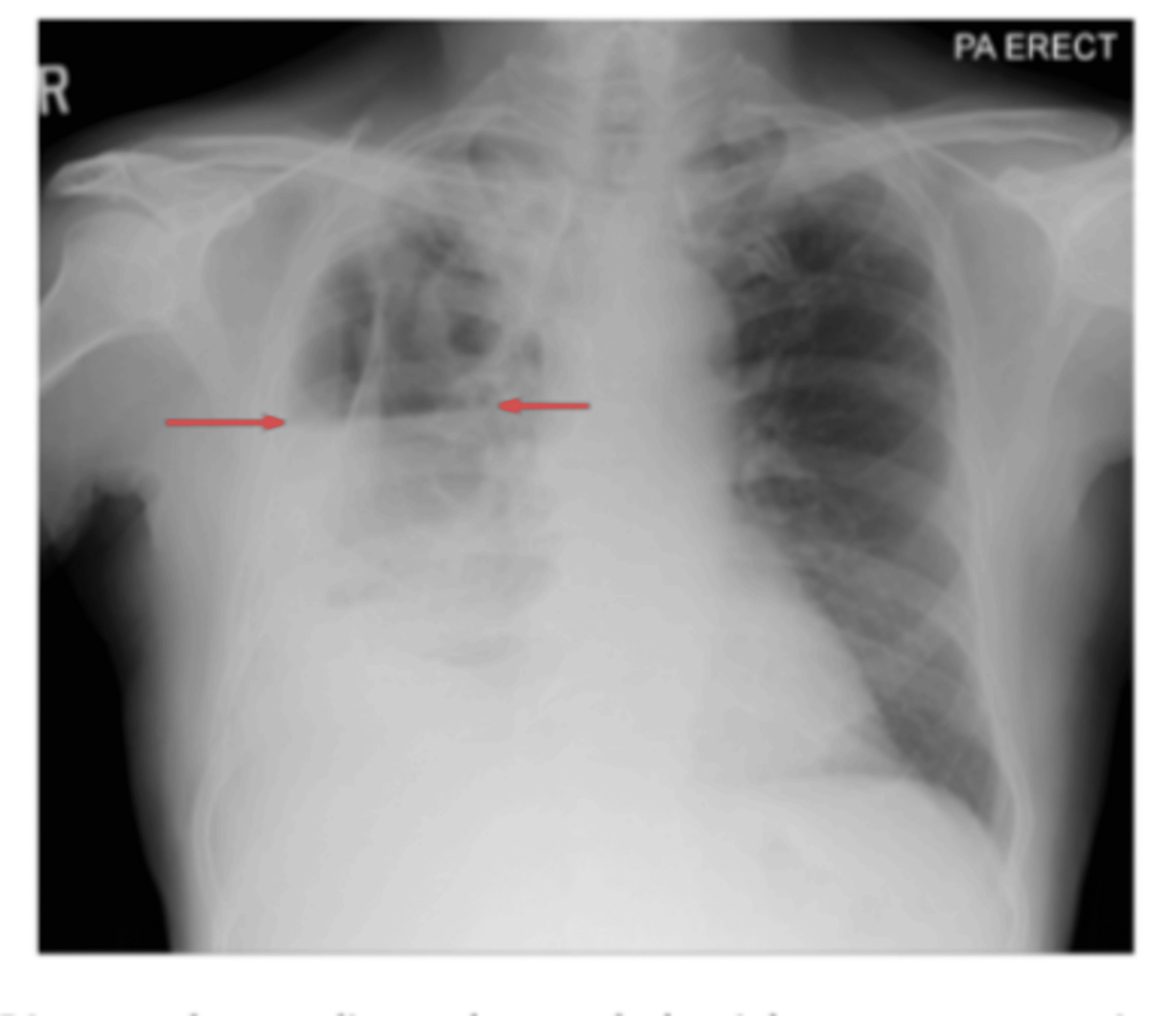
Posteroanterior (PA) erect chest radiograph showing a right upper-zone cavitary lung lesion with surrounding consolidation, suggestive of active infection. A right hydropneumothorax is also present, with an air-fluid level visible in the pleural cavity (arrows).

A chest radiograph showed a right-sided cavitary lesion with a hydropneumothorax (Figure 1), prompting pigtail catheter insertion, which drained exudative pleural fluid. Pleural fluid GeneXpert (Cepheid, Sunnyvale, California, USA) was positive for *M. tuberculosis*, and adenosine deaminase (ADA) was markedly elevated at 167 U/L (Table 2).

**Table 2:** Pleural fluid analysis. LDH: lactate dehydrogenase

Parameter	Result	Normal Range
Appearance	Milky	Pale yellow
Colour	Pale yellow	Straw colored
Protein	62.1 g/L	1-2 g/dL
Glucose	0.37 mmol/L	-
LDH	1883 IU/L	<200 IU/L
Body fluid culture	No growth	None
GeneXpert	Detected	-
Adenosine deaminase (ADA)	167 U/L	<40 U/L

**Figure 2:**
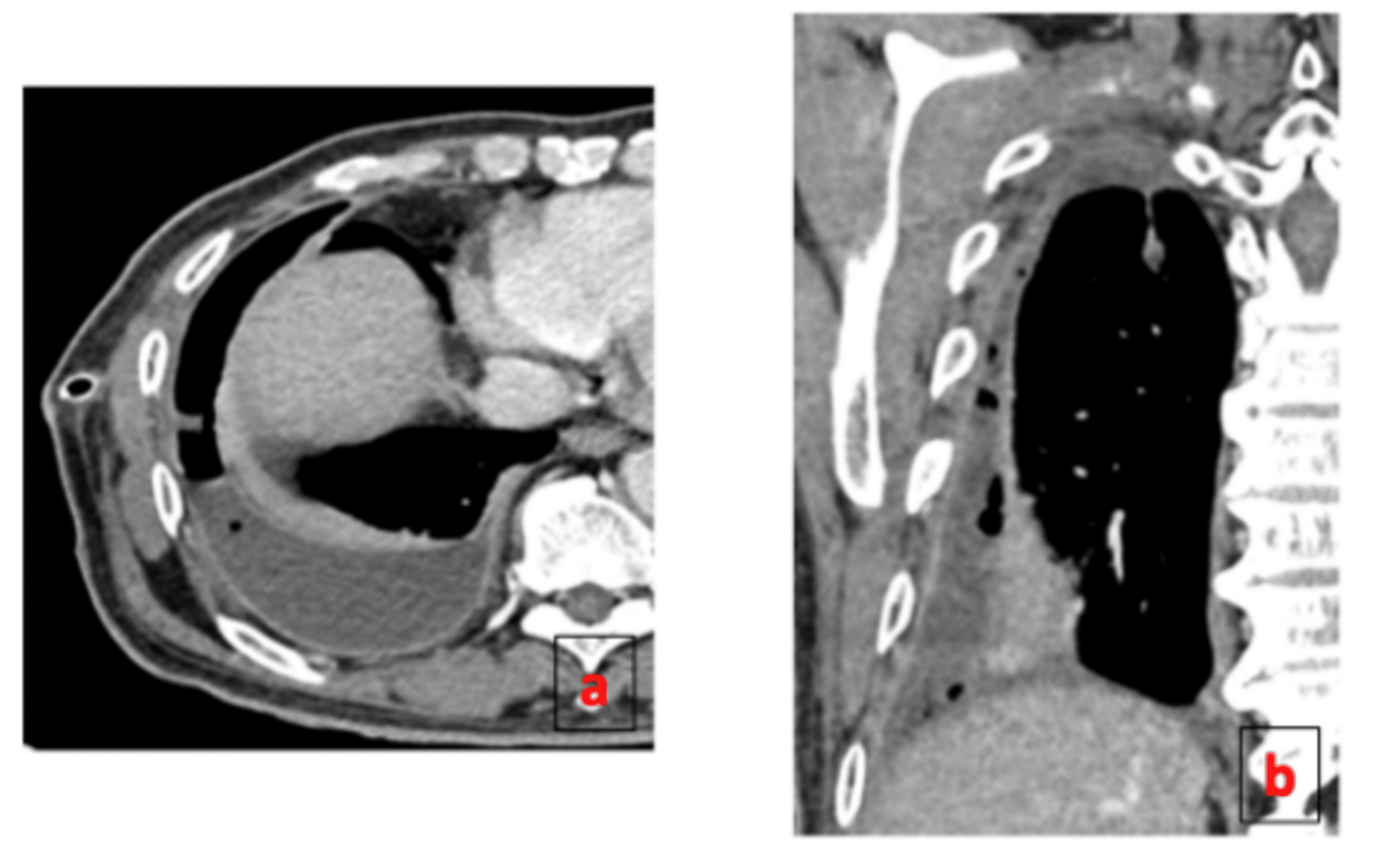
Contrast-enhanced CT of the thorax in axial (a) and coronal (b) views show right empyema with multiple air pockets within pleural cavity and enhanced parietal and visceral pleural giving rise to split pleura sign.

**Figure 3:**
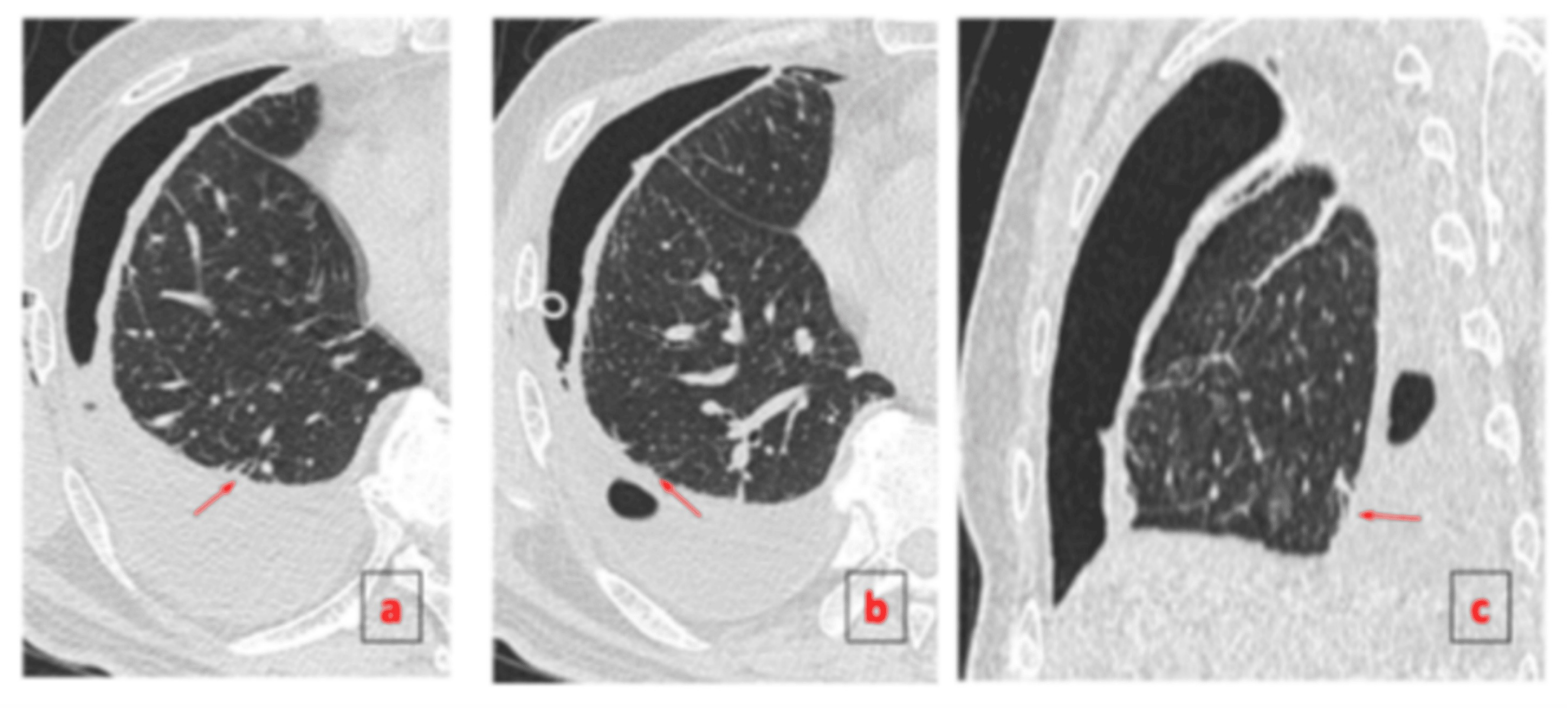
CT thorax in axial (a, b) and sagittal (c) views demonstrates a right hydropneumothorax. Multiple visceral pleural lining defects are noted, indicating direct communication between the pleural space and segmental bronchi of the right lower lobe (arrows).

**Figure 4:**
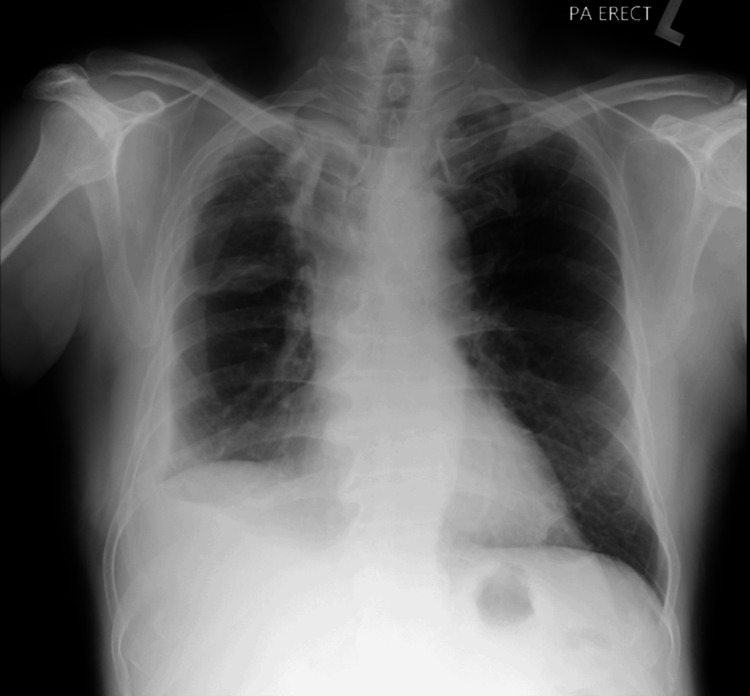
PA erect chest radiograph two weeks after ATT showed no recurrent pneumothorax. PA: posteroanterior; ATT: anti-tuberculosis therapy

**Figure 5:**
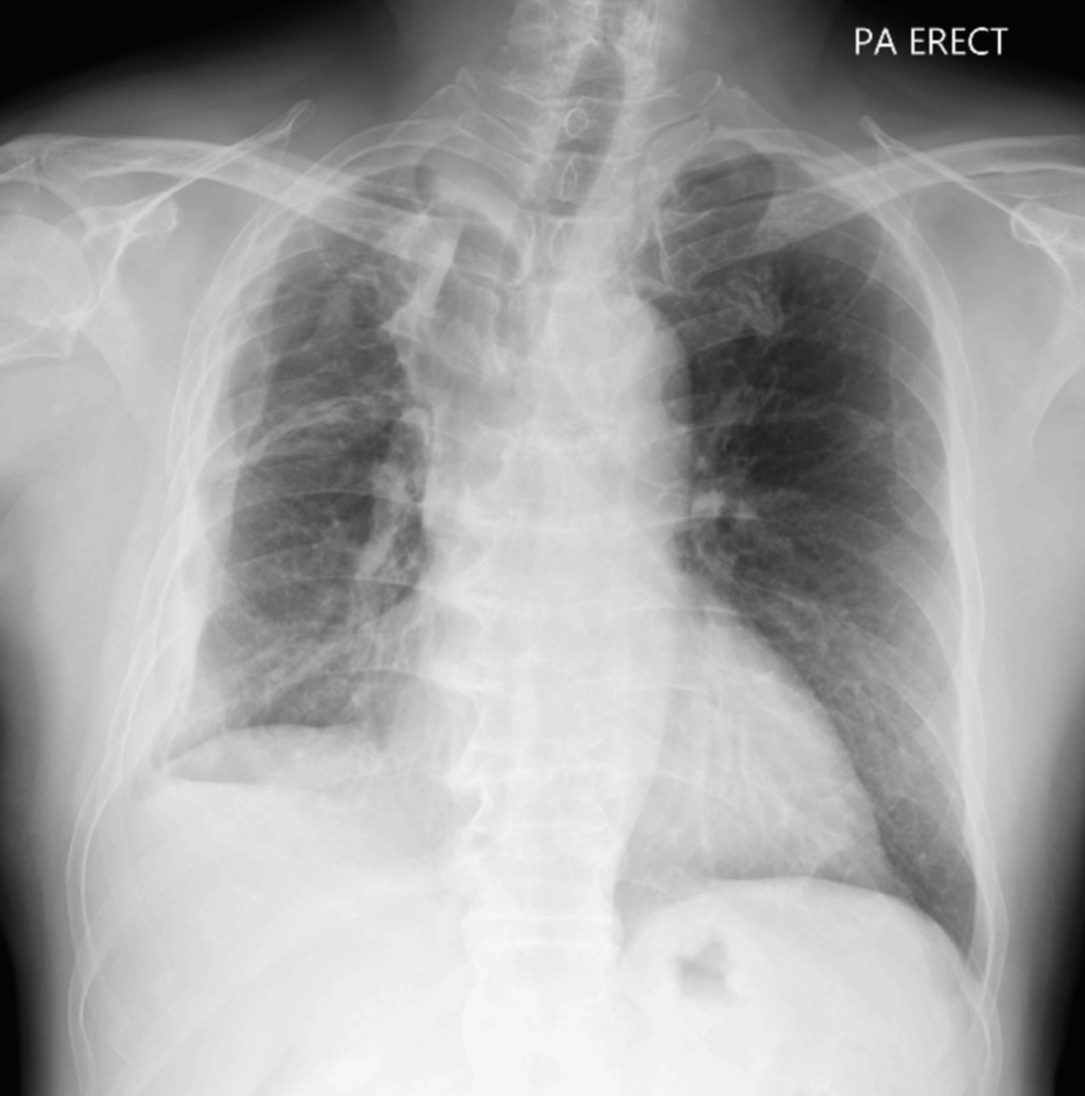
PA erect chest radiograph at six months after ATT showed no recurrent pneumothorax with fibrothorax. PA: posteroanterior; ATT: anti-tuberculosis therapy

The patient was started on standard first-line ATT consisting of rifampicin, isoniazid, pyrazinamide, and ethambutol. As the pneumothorax persisted beyond seven days despite appropriate catheter placement, an 18 Fr chest tube was inserted via blunt dissection into the right fifth intercostal space at the mid-axillary line and connected to an underwater seal, which demonstrated continuous bubbling. Contrast-enhanced CT thorax revealed multiple BPFs arising from segmental bronchi of the right lower lobe, consistent with active pulmonary TB and right-sided hydropneumothorax (Figures 2-3). Given the patient’s overall stable condition and absence of significant respiratory compromise, the cardiothoracic team decided on a non-operative strategy. ATT was continued, and the patient was closely monitored. Prior to discharge, the chest tube was connected to a portable one-way chest drainage system. Two weeks post-discharge, a follow-up chest radiograph showed resolution of the pneumothorax, allowing chest tube removal. Subsequent imaging showed no recurrence of pneumothorax (Figure 4). The patient remained clinically stable and tolerated ATT well throughout outpatient follow-up. Upon completion of six months of treatment, he remained well, had gained weight, and repeat imaging showed no recurrence of pneumothorax with fibrothorax (Figure 5).

## Discussion

TB remains a major global infectious disease, with pulmonary involvement being the most common presentation. BPF is an abnormal connection between the bronchial tree and pleural space, though it is a rare but potentially life-threatening complication [9]. This condition can lead to persistent air leaks and serious outcomes such as pneumothorax, empyema, subcutaneous emphysema, and respiratory failure [10]. Typically, BPF is associated with lung surgery or trauma, and those caused by TB are uncommon and under-reported in the literature.

The uniqueness of this case is the presence of multiple tuberculous BPFs complicated with hydropneumothorax in a patient with significant comorbidities, including poorly controlled diabetes mellitus and chronic smoking. Remarkably, the patient was successfully treated with a conservative approach. Most previously reported tuberculous BPFs involved single fistulas and frequently necessitated surgical intervention such as decortication, thoracoplasty, or window thoracostomy, particularly when complicated by infection or respiratory failure. Woldemariam et al. (2023) reported that most tuberculous BPF in Ethiopia require surgical treatment, mostly decortication and bronchial hole closure. The primary reason for the surgical intervention is a secondary bacterial infection [11]. In one case of tuberculous BPF, anti-TB drugs were given for two months with an installed chest tube but complicated with empyema secondary to multidrug-resistant *Klebsiella pneumoniae* and *Aspergillus fumigatus*. This patient required open window thoracostomy [12]. In contrast, our patient achieved complete resolution without surgery, relying on chest drainage, close monitoring, and ATT alone.

The diagnostic process also emphasises important learning points. Persistent air leaks beyond five to seven days should prompt further imaging, such as computed tomography to look for fistula [13], which in our case confirmed multiple segmental BPFs. The tests, such as pleural fluid ADA and GeneXpert, further strengthened the diagnosis of tuberculous aetiology [14]. Early recognition of BPF and rapid initiation of ATT were crucial in preventing progression to more severe complications such as empyema or sepsis.

Treatment of tuberculous BPF should be tailored to the individual. Although surgical intervention remains the standard for refractory or complicated cases, emerging evidence supports conservative management in select patients. Kasinathan and Pillai reported spontaneous closure of BPF with ATT alone [15], while Stewart and Boyd documented resolution after several months of conservative management [9]. Our case contributes to this limited but growing body of evidence, showing that even multiple BPFs with hydropneumothorax can resolve without invasive procedures, provided the patient remains clinically stable and infection is well-controlled.

This case highlights the importance of balancing operative risks against the potential for spontaneous healing, particularly in resource-limited settings or among patients with high surgical risk. It highlights the need for clinicians to remain vigilant for rare but serious complications of pulmonary TB and to consider conservative strategies when appropriate. Referral to a cardiothoracic surgeon is warranted in the presence of a persistent air leak, failure of medical therapy, recurrent infections, or a non-resolving pleural collection that may require decortication. In such circumstances, operative management may be necessary to achieve definitive closure and prevent further complications.

## Conclusions

Tuberculous BPF is a rare but serious complication of pulmonary TB that requires prompt recognition and tailored management. While surgical intervention is often necessary, especially in complicated or refractory cases, selected patients with stable clinical conditions may respond well to conservative treatment alongside ATT. In the present case, although there was no recurrent pneumothorax, the patient was left with residual fibrothorax. This highlights the importance of early diagnosis, appropriate imaging, and individualised care in achieving successful outcomes without the need for invasive procedures.
